# Coronary Vasospasm Masquerading as Coronary Artery Disease

**DOI:** 10.7759/cureus.86281

**Published:** 2025-06-18

**Authors:** Muhammad U Rana, Anuj Garg, Krishna Prasad Kurpad, Uday Kanakadandi, Muhammad Hasib Khalil, Talha Bin Farooq

**Affiliations:** 1 Internal Medicine, Carle Foundation Hospital, Urbana, USA; 2 Electrophysiology, Carle Foundation Hospital, Urbana, USA; 3 Cardiology, Carle Foundation Hospital, Urbana, USA

**Keywords:** cardiac arrhythmia, chest pain, coronary artery disease, vasospastic angina, ventricular tachycardia

## Abstract

Vasospastic angina, or Prinzmetal's angina, usually presents with chest pain. However, rare manifestations include ventricular fibrillation or cardiac arrest. Here, we discuss a case of a 78-year-old male who had recurrent episodes of syncope and was found to have ventricular fibrillation. He underwent coronary angiography and percutaneous intervention of the left anterior descending artery but continued to have syncope, which correlated with polymorphic ventricular fibrillation on an event monitor. Symptoms resolved with calcium channel blockers and nitrates, and the patient did not have further episodes of polymorphic ventricular tachycardia.

## Introduction

Vasospastic angina is a prevalent health problem, with 97,280 hospitalizations reported in the National Inpatient Sample database for the years 2002-2015. Hospitalization and in-hospital length of stay have increased over time, which is attributed to the overall burden of comorbidities in patients with vasospastic angina. This condition can be complex and difficult to diagnose and often requires provocative testing. However, making a definite diagnosis of vasospastic angina can improve quality of life, as shown in the CorMica (CORonary MICrovascular Angina) trial [1].

## Case presentation

A man in his 70s with a past medical history of paroxysmal atrial fibrillation, hypertension, pure hypercholesterolemia, benign prostatic hyperplasia, and chronic back pain due to spinal stenosis and degenerative disc disease presented to the emergency department with a complaint of passing out at home. He experienced his first episode of syncope while sitting in a chair. His wife noticed that while he was talking, he suddenly leaned back and passed out, remaining unresponsive for 20-30 seconds. She noted a strong carotid pulse during the episode. There were no associated symptoms like tongue bite, seizure-like activity, or loss of bowel or bladder control. The patient reported no preceding chest pain, lightheadedness, or dizziness. Three days prior, he had an episode of lightheadedness while walking, accompanied by burning chest pain that radiated to his throat and diaphoresis. The episode lasted about 10 minutes and resolved on its own. He also reported other episodes of lightheadedness and cold sweats while walking. He previously had a right hip arthroplasty in 2015. His medications include lisinopril 20 mg daily, tamsulosin 0.4 mg daily, apixaban 5 mg b.i.d., and simvastatin 40 mg daily.

On arrival, he had a BP of 116/73 mmHg, a heart rate of 63 bpm, a temperature of 97.4 °F, a respiratory rate of 14 breaths/min, and a saturation of 97% on room air. Physical examination revealed that his pulse was irregular, with normal S1 and S2, with no murmur or gallop. The neurological exam was normal, and lung sounds were clear.

Investigations

His CBC was normal. His comprehensive metabolic panel (CMP) showed creatinine 1.65 mg/dl, potassium 4.6 meq/dl, and magnesium 1.8 meq/dl. B-type natriuretic peptide test (BNP) was 88 pg/mL. Serial high sensitivity troponin at 0 and 2 hours was 7 ng/L and 7 ng/L, respectively. His D-dimer was 1.51. The CT angiography (CTA) chest did not show any pulmonary embolism or acute abnormalities. Baseline EKG showed atrial fibrillation. Transthoracic echocardiogram showed an ejection fraction of 55-60%; the aortic valve was mildly thickened, with no pericardial effusion and no wall motion abnormalities.

Differential diagnosis

The differential diagnosis included tachyarrhythmia, bradyarrhythmia, seizure, and transient ischemic attack (TIA). Given that the wife mentioned that the patient had a strong pulse and there was very minimal elevation in troponin, ischemic etiology-related arrhythmia was considered less likely.

Treatment

**Figure 1:**
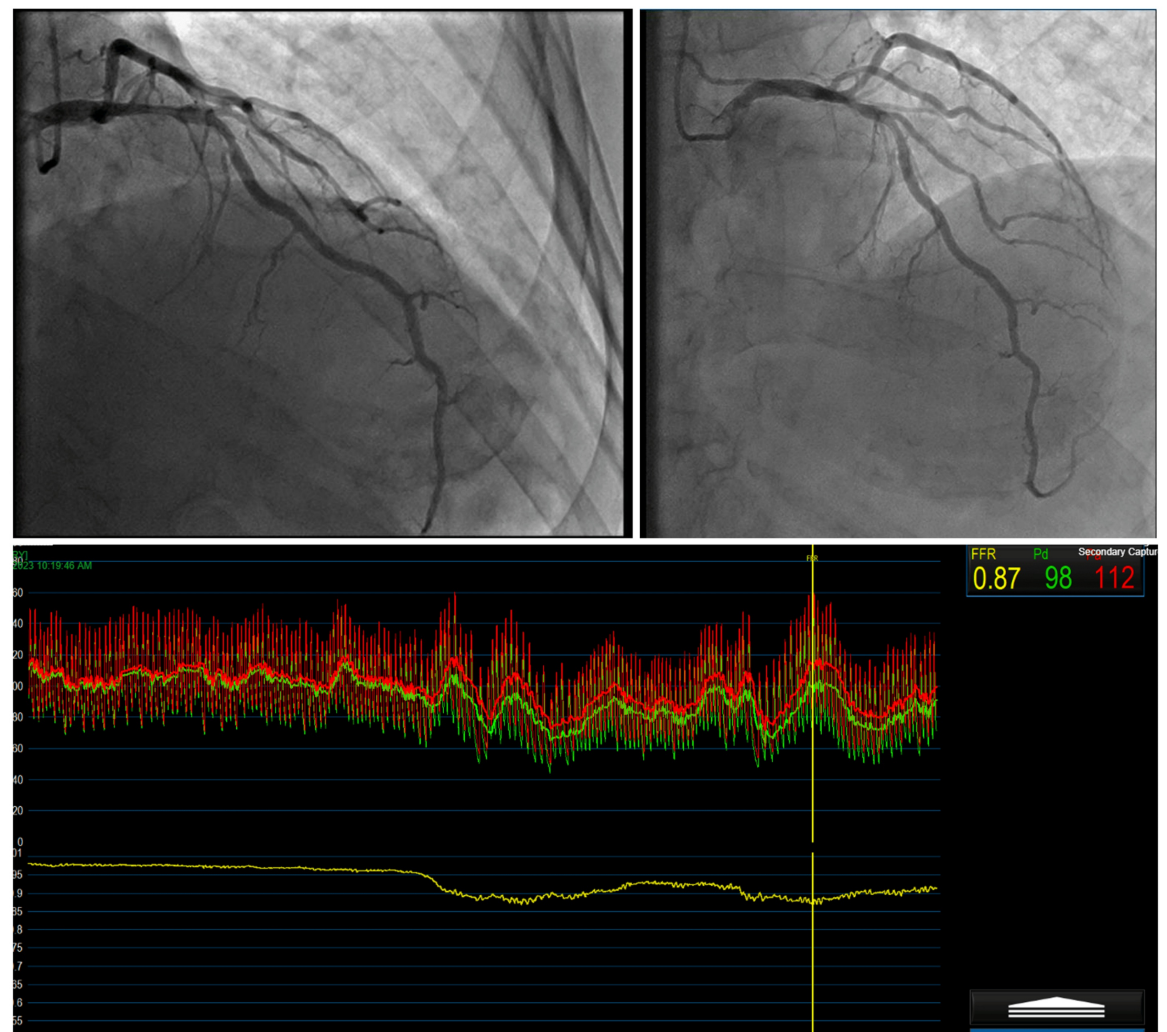
The top left image shows 60-70% stenosis in the proximal to mid LAD artery with FFR 0.87. The top right image shows 50% stenosis in the LCX coronary artery with an IFR of 0.96 and 30-40% stenosis in the distal right coronary artery. The bottom image shows an FFR of 0.87, indicating stenosis of the LAD artery, and an IFR of 0.96, indicating stenosis of the LCX artery is not hemodynamically significant. In the pressure waveforms, red represents aortic pressure (Pa) and yellow represents distal coronary artery pressure (Pd). LAD: left anterior descending; FFR: fractional flow reserve; IFR: instantaneous wave-free ratio; LCX: left circumflex

An event recorder was recommended, and the patient was discharged. However, before the event recorder could be placed, the patient presented back to the hospital with another episode of syncope while sitting in a chair. This time, the patient had chest pain with syncope, central and radiating down to the arms and back. Figure 1 shows the results of his left heart catheterization and coronary angiography. Medical management was recommended, and the patient was discharged.

Outcome and follow-up

**Figure 2:**
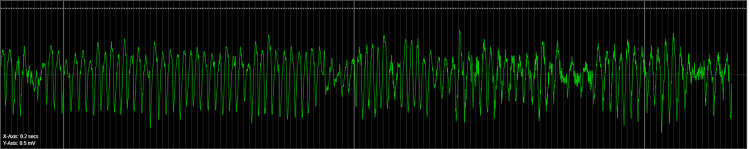
Patient's cardiac telemetry shows polymorphic ventricular tachycardia

The patient presented to the hospital again two weeks later with another episode of passing out. At this time, he was wearing an event monitor, which showed polymorphic ventricular tachycardia, as seen in Figure 2.

Given the patient's moderate disease, it was considered that ischemia might be contributing to the symptoms. The decision was made to revascularize the mid-LAD stenosis, and the patient underwent percutaneous coronary intervention (PCI) with the placement of a drug-eluting stent. He was discharged on a LifeVest.

**Figure 3:**
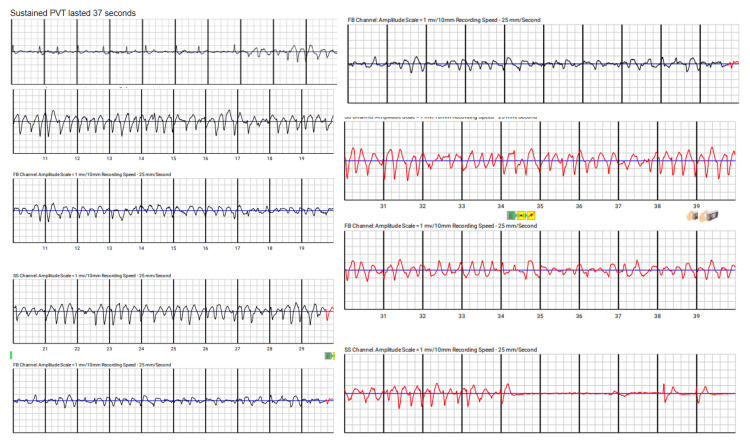
Polymorphic sustained ventricular tachycardia lasting 37 seconds from LifeVest telemetry strips

Two weeks later, the patient was seen in the cardiology clinic and reported feeling better, though he mentioned a few episodes of chest pain that subsided with sublingual nitroglycerin. A few days after that visit, the electrophysiology clinic was alerted to episodes of polymorphic ventricular tachycardia. The patient felt lightheaded and had midsternal chest pain, and his LifeVest alerted him twice before he removed it. Device interrogation showed 37 seconds of polymorphic ventricular tachycardia and failed device shocks as seen in Figure 3.

After considering the risks versus benefits, an implantable cardioverter defibrillator (ICD) was recommended, and the patient agreed. He subsequently underwent an ICD placement. The patient was also started on verapamil and long-acting nitrate.

## Discussion

Coronary vasospasm, initially described by Dr. Myron Prinzmetal in 1959, is characterized by transient constriction of the coronary arteries, leading to episodic chest pain (angina) and, in rare cases, more severe manifestations such as ventricular fibrillation or cardiac arrest, mainly by causing ischemia [2]. This case highlights a 78-year-old male with recurrent syncope and ventricular fibrillation, ultimately diagnosed with vasospastic angina masquerading as coronary artery disease.

Our patient presented with recurrent episodes of syncope, initially mistaken for obstructive coronary artery disease due to the presence of moderate stenosis on coronary angiography. Despite the percutaneous intervention, the patient continued to experience syncope and ventricular fibrillation, which were ultimately resolved with calcium channel blockers and nitrates, confirming the diagnosis of coronary vasospasm.

This case underscores the diagnostic challenge of differentiating between coronary vasospasm and obstructive coronary artery disease. Traditional markers and angiographic findings may not always clearly distinguish between the two, particularly in the presence of moderate coronary artery stenosis, as was the case with our patient. The decision to revascularize the mid-LAD stenosis was based on the assumption that ischemia was contributing to the patient’s symptoms. However, the continued episodes of polymorphic ventricular tachycardia despite revascularization highlighted the need for a different therapeutic approach.

Coronary vasospasm has been implicated in a significant proportion of out-of-hospital cardiac arrests, with recent studies indicating that 7-11% of such events are related to this condition [3]. Survivors of out-of-hospital cardiac arrests due to coronary vasospasm face a high risk of recurrence, necessitating thorough evaluation and management strategies to prevent future episodes. Calcium channel blockers and nitrates are known to mitigate vasospastic episodes by promoting vasodilation and preventing coronary artery constriction [4,5].

The therapeutic approach in this case was adjusted based on evolving clinical findings, ultimately leading to the placement of an ICD after persistent episodes of polymorphic ventricular tachycardia. This step was crucial in providing a safety net for the patient, given the high recurrence rate of arrhythmias associated with coronary vasospasm.

## Conclusions

Diagnosing and managing coronary vasospasm is complex, particularly when it presents with symptoms that mimic obstructive coronary artery disease. Vasospastic angina in patients should be considered in patients with recurrent syncope and ventricular arrhythmias, even when moderate coronary artery stenosis is present. Calcium channel blockers and nitrates show efficacy in resolving symptoms in managing coronary vasospasm. The role of an ICD in preventing sudden cardiac death in such high-risk patients is underscored. Further research is needed to better stratify patients at risk for coronary vasospasm and to develop standardized treatment protocols.
